# Force quantification and simulation of pedicle screw tract palpation using direct visuo-haptic volume rendering

**DOI:** 10.1007/s11548-020-02258-0

**Published:** 2020-09-21

**Authors:** Esther I. Zoller, Balázs Faludi, Nicolas Gerig, Gregory F. Jost, Philippe C. Cattin, Georg Rauter

**Affiliations:** 1 BIROMED-Lab, Department of Biomedical Engineering, University of Basel, Basel, Switzerland; 2 CIAN, Department of Biomedical Engineering, University of Basel, Basel, Switzerland; 3 Spinale Chirurgie, Spitalzentrum Biel, Biel, Switzerland

**Keywords:** Haptic rendering, Virtual reality, CT, Medical simulation, Human robot interaction

## Abstract

**Purpose:**

We present a feasibility study for the visuo-haptic simulation of pedicle screw tract palpation in virtual reality, using an approach that requires no manual processing or segmentation of the volumetric medical data set.

**Methods:**

In a first experiment, we quantified the forces and torques present during the palpation of a pedicle screw tract in a real boar vertebra. We equipped a ball-tipped pedicle probe with a 6-axis force/torque sensor and a motion capture marker cluster. We simultaneously recorded the pose of the probe relative to the vertebra and measured the generated forces and torques during palpation. This allowed us replaying the recorded palpation movements in our simulator and to fine-tune the haptic rendering to approximate the measured forces and torques. In a second experiment, we asked two neurosurgeons to palpate a virtual version of the same vertebra in our simulator, while we logged the forces and torques sent to the haptic device.

**Results:**

In the experiments with the real vertebra, the maximum measured force along the longitudinal axis of the probe was 7.78 N and the maximum measured bending torque was 0.13 Nm. In an offline simulation of the motion of the pedicle probe recorded during the palpation of a real pedicle screw tract, our approach generated forces and torques that were similar in magnitude and progression to the measured ones. When surgeons tested our simulator, the distributions of the computed forces and torques were similar to the measured ones; however, higher forces and torques occurred more frequently.

**Conclusions:**

We demonstrated the suitability of direct visual and haptic volume rendering to simulate a specific surgical procedure. Our approach of fine-tuning the simulation by measuring the forces and torques that are prevalent while palpating a real vertebra produced promising results.

**Electronic supplementary material:**

The online version of this article (10.1007/s11548-020-02258-0) contains supplementary material, which is available to authorized users.

## Introduction

Pedicle screws are commonly used in spinal fusion surgeries to treat congenital spine deformities as well as traumatic or degenerative spine conditions. Before placing a pedicle screw, the posterior elements of the spine are exposed. At the entry point, the cortical bone of the vertebra is then breached with a burr and the pedicle screw tract created with an awl or a drill. Next, the bony walls of the pedicle are palpated for their integrity, i.e., the screw tract is checked for anterior, medial, lateral, superior, or inferior breaches with a ball-tipped probe or similar device. Finally, the screw is inserted and its placement is verified via imaging [11]. However, placing these screws correctly is not trivial. Several meta-analyses found the mean pedicle screw placement accuracy, i.e., the percentage of correctly placed pedicle screws without breaches, to be about 80–90% [12, 17, 25]. The main reasons for pedicle screw misplacement are most likely that the pedicle is not visible to the surgeon [4] and that the morphology of the pedicles varies between patients [7]. The use of navigation techniques has been shown to improve pedicle screw placement accuracy [1, 9, 12, 17, 24, 25]. However, reported mean placement accuracy still varies between 92.4 and 97.3% [1, 12, 17, 25], possibly due to limitations such as calibration errors, instrument bending, or non-rigid connections between reference and surgical site [8]. It can thus be assumed that even with the help of modern navigation tools, roughly 1 out of 20 pedicle screws is misplaced. Misplaced pedicle screws can cause several problems such as neurological, vascular, or visceral damage [8]. The consequences include radicular pain, sensorimotor deficits, paralysis, or even death of the patient [1, 8]. If the misplacement is observed intraoperatively (e.g., by palpating the screw tract with a ball-tipped probe or imaging techniques), the screw can be replaced during the same surgery and a damaged dural or vascular structure sutured or embolized. Postoperative complications attributable to screw misplacement might require surgical revision and possibly an extension of the fixation to additional levels [8].

Manual palpation of the drilled screw tract using a probe is a crucial step to prevent misplaced pedicle screws [5, 11, 14, 23, 26]. However, studies investigating the reliability of this technique concluded that surgeons do not excel in correctly identifying pedicle breaches using a ball-tipped probe [5, 14, 23]. To identify such breaches, the surgeon needs to interpret the perceived haptic feedback correctly, which is a skill that takes years of surgical practice to develop. It is thus not surprising that the surgical experience and level of training have been identified as critical factors for the reliability of manual pedicle screw tract palpation [14], as well as for the correct placement of pedicle screws [7, 10].

One possibility to foster the development of the haptic skills of surgeons is the use of simulators. Simulators allow trainees to acquire skills in a no-risk environment through repeated practice in varied scenarios [16]. Virtual simulators in particular make it simple to record collision forces and tool paths for an objective quantification of the task performance. However, even though it has been stated that developers of virtual surgical simulators should pay close attention to providing realistic haptic feedback [3, 22], accurately simulating the haptic sensations of surgical procedures remains a major challenge [16].

In previous work, we presented a method for direct visual and haptic volume rendering of medical data sets in virtual reality (VR) [6]. Unlike previous approaches [2, 13, 21], our method allows rendering the patient-specific anatomy without relying on any mesh or surface generation, guarantees a passive, continuous force field, and showed stable haptic feedback even for stiff objects such as bony structures. The goal of this work was to test the feasibility of the above-mentioned approach to simulate the palpation of a drilled pedicle screw tract with a ball-tipped probe. As it has been suggested that the fidelity of the haptic experience provided by surgical simulators should be pursued more than their graphical accuracy [3], we first performed an experiment to quantify the interaction forces and torques perceived by a surgeon while palpating a real pedicle screw tract, which is presented in “Force and torque quantification” section. The implementation and validation of our visuo-haptic VR simulation of pedicle screw tract palpation is described in “Simulation of pedicle screw tract palpation” section.

## Force and torque quantification

To define the requirements for the simulation, we quantified the forces and torques felt by a surgeon while palpating a real pedicle screw tract with a ball-tipped probe.

### Methods

#### Specimen and apparatus

As a substitute for human vertebrae, we obtained a fresh lumbar spine of a young boar from a local hunter. Using a hand-held electric drill with a 3 mm metal drill bit, we created screw tracts in several boar vertebra pedicles. For our measurements, we chose a vertebra with a fully intact screw tract in the right pedicle.

**Fig. 1:**
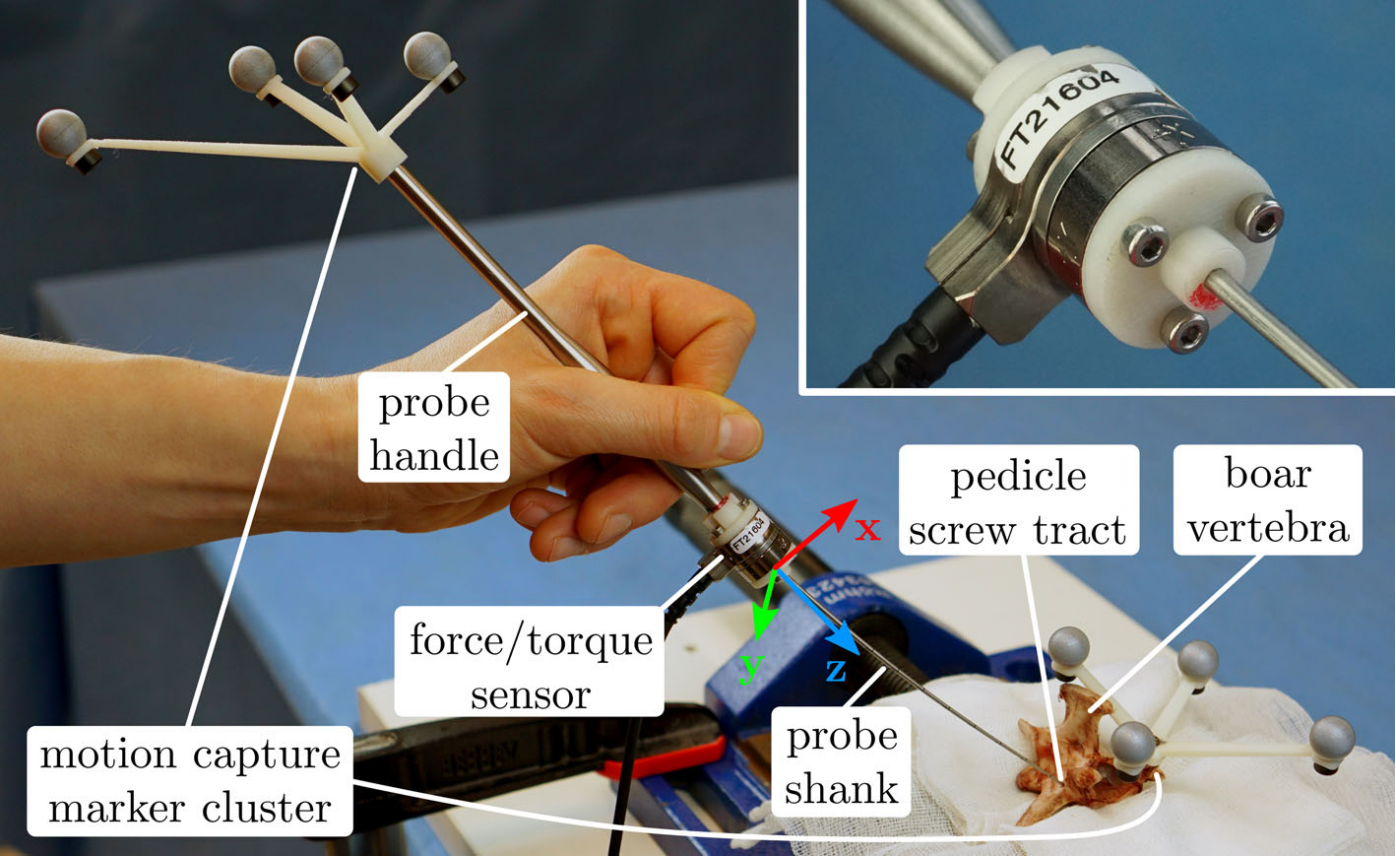
Setup for the force/torque quantification measurements. The Nano17 force/torque sensor (ATI Industrial Automation, Apex, NC, USA) was mounted co-axially between the separated shank and handle of the pedicle probe (see close-up). Due to elastic probe bending, the probe shank is not necessarily parallel to the *z*-axis during palpation. Both the probe and vertebra poses were tracked by the motion capture system

We used a straight ball-tipped pedicle probe (Cat. No. 2750-10-140, DePuy, Le Locle, Switzerland), which weighed 46.4 g. To measure the prevalent forces and torques while palpating the pedicle screw tract with the pedicle probe, we equipped it with a 6-axis Nano17 force/torque sensor (ATI Industrial Automation, Apex, NC, USA) with SI-12-0.12 calibration, which weighed 9.07 g (see Fig. 1). The forces and torques were recorded at a rate of 100 Hz. Since we were unsure about the maximum torques to be expected during pedicle probing, we initially also conducted measurements with a second force/torque sensor (see Online Resource 1).

In addition to measuring the forces and torques, we also recorded the poses of the pedicle probe and the boar vertebra during the experiment. For this, we used four Oqus 700+ motion capture cameras (Qualisys AB, Gothenburg, Sweden). All cameras were operated with a capture rate of 100 Hz. The motion capture system was calibrated according to the instructions of the manufacturer. The maximum of the average residuals of the cameras in the used calibrations was 0.446 mm. We designed and 3D printed two motion capture marker clusters comprised of four passive reflective markers each. One of the marker clusters was attached to the end of the probe and the second one to the left superior articular process of the vertebra (see Fig. 1).

#### Experimental procedure

We recruited two neurosurgeons to quantify the prevalent forces and torques while palpating a real pedicle screw tract. While surgeon 1 was very experienced with pedicle screw placements (16 years of experience), surgeon 2 was relatively inexperienced (3 years of experience). We asked the surgeons to palpate the pedicle screw tract with the instrumented probe as they would palpate a human pedicle during surgery (see Fig. 1 and Online Resource 2). We recorded the palpation with each hand, because the surgeons reported to perform this procedure with either hand depending on the situation. For the first surgeon, we performed the measurements three times with the left (preferred) and right hand each. For the second surgeon, we recorded the palpation three times with the preferred (left, non-dominant) hand and once with the non-preferred hand.

#### Data analysis

The motion capture system allowed us to track the pose of the pedicle probe marker cluster relative to the boar vertebra marker cluster during our force and torque measurements. However, for our simulation, we were interested in the motion of the sensor attached to the pedicle probe relative to the screw tract in the vertebra. Thus, we also had to determine the relative pose of the sensor with respect to the pedicle probe marker cluster and of the screw tract with respect to the vertebra marker cluster. For this, we used a nanotom^®^ m (phoenix|x-ray, GE Sensing & Inspection Technologies GmbH, Wunstorf, Germany) to perform two high-resolution CT scans: one of the boar vertebra with the screw tract and the attached marker cluster and one of the pedicle probe handle with the attached marker cluster and Nano17 force/torque sensor. Using the motion capture data and the CT scans which had a voxel size of $\big (69.5,\;69.5,\;69.5\big )^\intercal \,\upmu \text {m}$, we reconstructed the pose of the force sensor relative to the pedicle screw tract for each palpation trial (for details see Online Resource 1). We synchronized the motion capture data and the force/torque measurements by aligning the force peaks of five subsequent taps of the probe tip against a solid surface to the respective motion peaks.

### Results

**Fig. 2:**
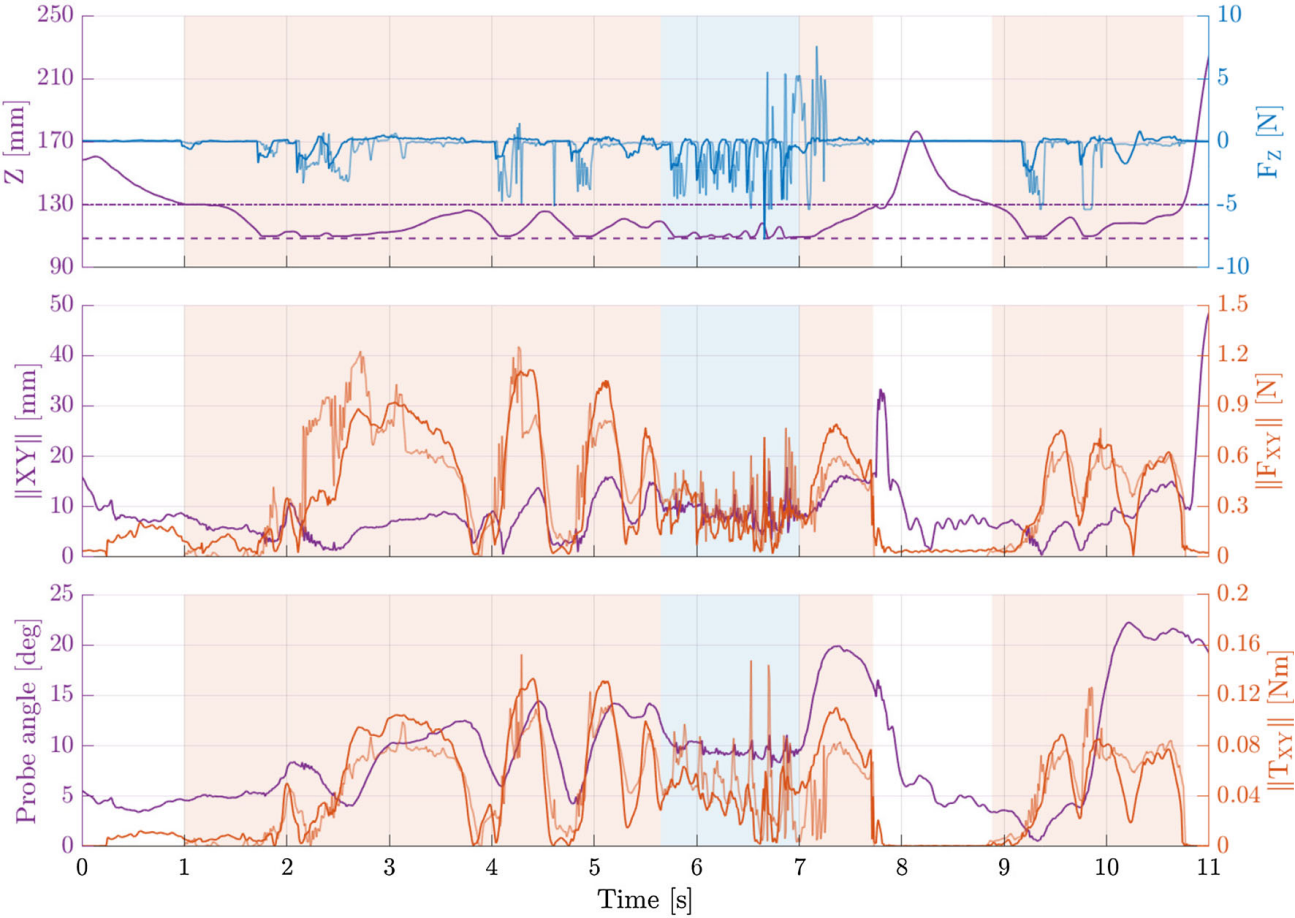
Recorded force/torque data from an exemplary palpation trial of the more experienced surgeon (surgeon 1). The palpation of the sides and the anteriormost end of the screw tract are indicated with orange and blue backgrounds, respectively. Top: Distance between sensor and screw tract entrance along the screw tract axis (purple), the measured longitudinal forces (dark blue), and the forces simulated based on the recorded motion data (light blue). The horizontal lines indicate where the pedicle probe tip enters (dash-dot) and reaches the anteriormost end (dashed) of the screw tract. Middle: Sensor distance to the screw tract axis (purple), the measured transverse forces (dark orange), and simulated forces (light orange). Bottom: Angle between screw tract and probe handle (purple), the measured bending torques (dark orange), and simulated torques (light orange)

The motion of the force/torque sensor attached to the pedicle probe relative to the pedicle screw tract in the vertebra and the corresponding forces/torques are shown in Fig. 2 for an exemplary trial.

**Fig. 3:**
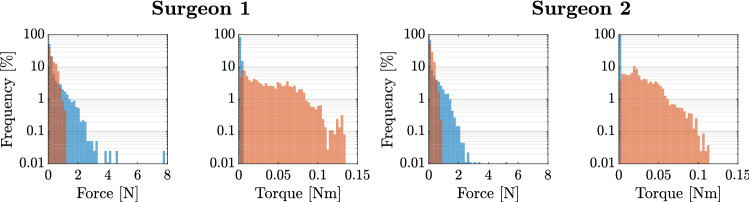
Measured force and torque distribution during all boar vertebra palpation trials of the two surgeons. The blue histograms visualize the forces along the longitudinal axis of the pedicle probe handle and the twisting torques. The orange histograms visualize the forces in the plane perpendicular to the longitudinal axis of the pedicle probe handle and the bending torques. For both surgeons, we concatenated the recorded force and torque sequences from all of their trials and computed the histograms of the resulting sequences

The recorded forces and torques over all trials, grouped by surgeon, are displayed in Fig. 3.

The mean, standard deviation, and maximum of all recorded forces and torques above 0.01 N and 0.001 Nm, respectively, are shown in Table 1.

## Simulation of pedicle screw tract palpation

To provide a training platform for pedicle screw tract palpation, we developed a visuo-haptic VR simulator, which does not require manual processing or segmentation of the volumetric medical data.

### Methods

#### Hardware

**Table 1:** Mean, standard deviation, and maximum of the measured forces and torques during palpation of the vertebra screw tract

	$F_{xy}$ (N)			$F_{z}$ (N)			$T_{xy}$ (Nm)			$T_{z}$ (Nm)		
	$\mu$	$\sigma$	max	$\mu$	$\sigma$	max	$\mu$	$\sigma$	max	$\mu$	$\sigma$	max
Surgeon 1	0.28	0.24	1.12	0.31	0.47	7.78	0.037	0.029	0.134	0.002	< 0.001	0.005
Surgeon 2	0.19	0.15	0.89	0.23	0.36	5.14	0.028	0.020	0.113	0.001	< 0.001	0.002

Forces below 0.01 N and torques below 0.001 Nm have not been considered

**Fig. 4:**
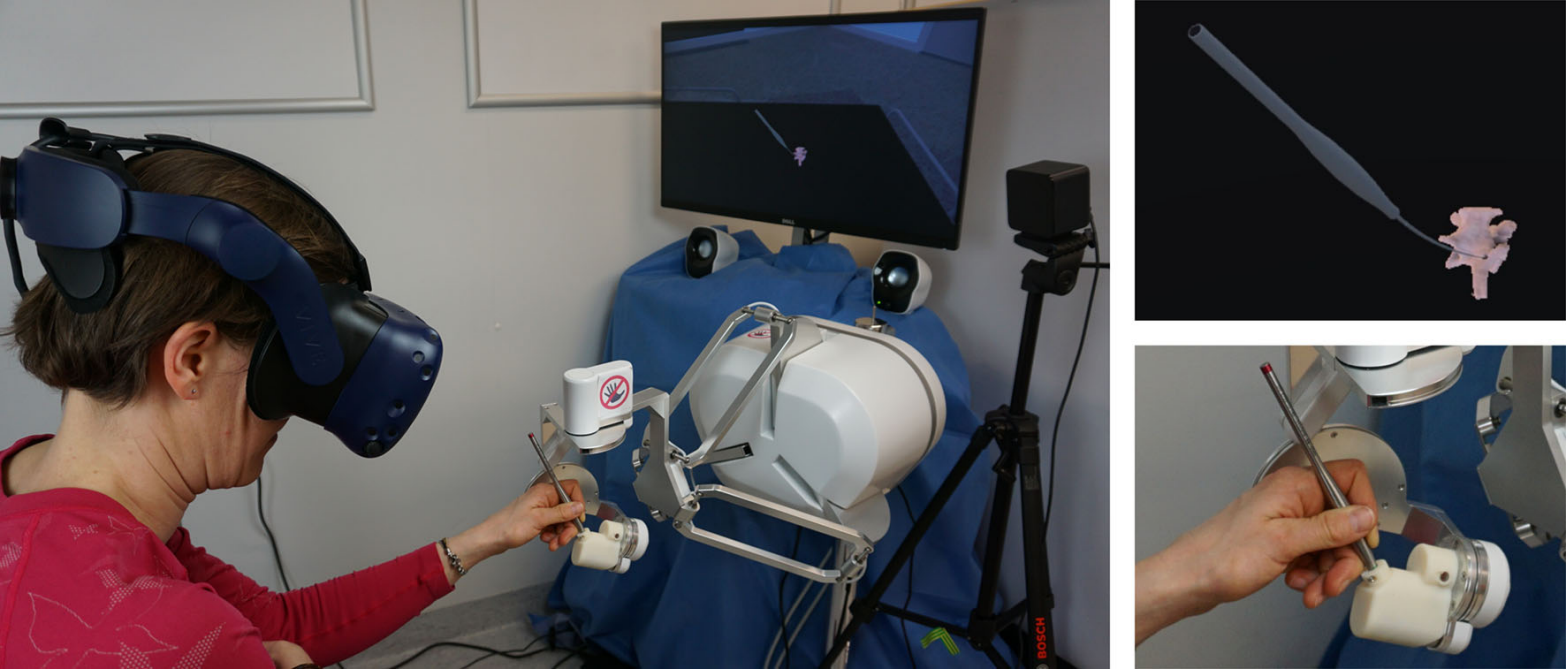
Left: Simulation setup. A user interacts with the virtual environment through the haptic input device. The virtual environment is visually perceived through the head-mounted display. Upper right: Close-up of the virtual environment: the pedicle probe is used to palpate the pedicle screw tract in the boar vertebra. Lower right: Close-up of the pedicle probe handle mounted to the lambda.6 haptic input device

Our hardware setup for the simulation consisted of an HTC Vive Pro head-mounted display (HMD) with a Lighthouse 1.0 tracking system (both HTC, New Taipei City, Taiwan) and a customized six degrees of freedom lambda.6 haptic input device (Force Dimension, Nyon, Switzerland) (see Fig. 4).

The lambda.6 is a high-end haptic device with low inertia and low friction, thus allowing a high degree of transparency, i.e., the undesired interaction forces present while using the device are very low [19]. Both the HMD and the haptic device were controlled by the same computer (HP Z640 Workstation, Intel Xeon E5-2630 CPU @ 2.2GHz, Nvidia GTX 1080 GPU, 16 GB RAM, Windows 10).

#### Visualization

For the visualization of the vertebra, we used the high-resolution CT scan of the boar vertebra and the VR application SpectoVR (University of Basel, Basel, Switzerland). This software is capable of loading and displaying volumetric medical images provided in standard DICOM format. It implements an efficient ray-marching-based volume renderer optimized to render 90 frames per second per eye. A transfer function maps the grayscale values of the raw voxel data (Hounsfield values in the case of a CT scan) to color and opacity values.

#### Haptic rendering

Since our approach does not use an explicit definition of collision surfaces, we could not apply conventional mesh-based collision detection methods. Instead, we defined the haptic forces based on the visualized voxel opacity values $\alpha$ of the high-resolution CT data of the boar vertebra. First, we blurred the voxel opacities with a standard Gaussian kernel (see Online Resource 1). We empirically found a blur kernel with a size of 15 voxels and $\sigma = 1$ voxel in all three dimensions to provide an acceptable compromise between keeping the bone tissue stiffness within the capabilities of our haptic device and losing too much haptic detail. Then, we computed the negative gradient $\varvec{g}$ of the blurred voxel opacities at discrete positions with the same resolution as the original data set:

1 $$\begin{aligned} \varvec{g} = -\nabla (\omega * \alpha ). \end{aligned}$$

To evaluate $\varvec{g}$ at any position $\varvec{p} = (x,y,z)$, we used tricubic interpolation with a Catmull–Rom interpolation kernel. We defined the haptic force at any given interaction point $\varvec{p_i} = (x_i,y_i,z_i)$ as

2 $$\begin{aligned} \varvec{F_i} = c_f \varvec{g}(\varvec{p_i}), \end{aligned}$$

where $c_f > 0$ is a constant conversion factor. Finally, we converted $\varvec{F_i}$ to a force $\varvec{F_{i}^{*}}$ and torque $\varvec{\tau _{i}^{*}}$ at the device end-effector according to the law of the lever.

Computing the haptic forces only for one interaction point is sufficient to palpate the surface of a structure [6]. However, this is not enough for our current application, because there can easily be multiple contact points between the shank of the pedicle probe and the surrounding pedicular bone. As only the front end of the probe shank should be in contact with the vertebra, we defined *n* haptic interaction points at fixed intervals starting from the tip of the probe shank. The forces and torques from all haptic interaction points were summed up and scaled with the constant factors $s_f$ and $s_t$:

3 $$\begin{aligned} \varvec{F^{*}} = s_f \sum _i^n\varvec{F_{i}^{*}}, \varvec{\tau ^{*}} = s_t \sum _i^n\varvec{\tau _{i}^{*}}. \end{aligned}$$

Finally, these forces and torques were sent to the haptic device for rendering.

#### Iterative pedicle probe bending

The mechanical properties of the pedicle probe cause its shank to bend during palpation of the pedicle screw tract. To simulate this bending behavior, which is important for a realistic virtual replication of the palpation procedure, we used a simplified cantilever beam model with one fixed end (shank-handle interface) and one free end (probe tip). Based on rough estimations from our first experiment, we expected relatively small deflections of the tip of up to 3 cm. Therefore, we only considered deflections in a plane perpendicular to the longitudinal (*z*) axis of the probe. The deflection $\varvec{v}(z)$ of the beam with the force $\varvec{F}$ being applied to the free end is:

4 $$\begin{aligned} \varvec{v}(z) = \frac{\varvec{F}z^2}{6EI}(3L - z), \end{aligned}$$

where *z* is the distance from the fixed end of the beam, $\varvec{F}$ is the applied force, *E* is the Young’s modulus of the beam material, *I* is the area moment of inertia of the beam, and *L* is the total length of the beam. We assumed $E = 195\,\text {GPa}$, which corresponds to corrosion-resistant steel. The total length *L* of our pedicle probe shank was 130 mm. The radius of the pedicle probe shank increased from 0.4 mm at the tip to 1.0 mm at the handle-shank interface. For simplicity, we assumed a constant average radius of 0.7 mm, resulting in an area moment of inertia $I = \frac{1}{4}\pi r^4 = 0.189\,\text {mm}^4$.

The procedure we used to update the probe bending at every simulation time step is explained in the following. First, we applied the deflection computed during the previous time step to the current probe pose and computed the probe’s resistive force against this bending for each interaction point according to Eq. 4. Additionally, we determined the haptic force at the position of each interaction point according to Eq. 2. The sum of the resistive force and the haptic force was then converted to a force at the probe tip for each interaction point. The forces at the probe tip from all *n* interaction points were summed up and the probe’s bending was updated in the direction of the resulting force. These steps were repeated until the resulting force sum was smaller than 0.001 N (quasistatic equilibrium) or a maximum number of iterations $i_{\max }$ was reached. The final shape was then used to calculate the interaction forces and torques as described in “Haptic rendering” section, which were then sent to the haptic device for rendering.

#### Parameter tuning

Several parameters in our simulation had no direct relation to physical properties of the simulated objects and therefore had to be determined empirically. We tuned these parameters such that the simulated forces and torques approximated the measured ones. We used the recorded data from one exemplary palpation performed by the more experienced surgeon from the force/torque quantification experiments described in “Force and torque quantification” section. The motion of the force/torque sensor was interpolated to 4 kHz using cubic splines to match the maximum update rate of the haptic device and used as an input for the simulation. We knew that during the force/torque quantification trial, the probe tip entered the screw tract. To achieve this behavior in the offline simulation as well, we introduced a manually determined offset to the sensor position of $\big (0,\;4.5,\;0.5\big )^\intercal \,\text {mm}$. This was necessary as unlike the virtual probe, our real probe shank was not perfectly straight and the accuracy of the calculated probe sensor position was limited by the motion capture accuracy and the measurements in the CT scans.

We ran the offline simulation of the recorded probe motion with different parameters and visually compared the computed forces and torques to the ones measured by the force/torque sensor. We determined $c_f = 5\,\hbox {Nm}$, $s_f = 0.3$, and $s_t = 0.3$ to result in the best approximation. We found $n=3$ interaction points (9 mm apart) to be sufficient, since the relatively high bending radius of the probe and the cylindrical shape of the screw tract made it unlikely to have more than three relevant contact points. For the maximum iteration count, we found $i_{\max } = 1000$ to provide a good trade-off between not reaching an equilibrium point and compromising the update rate of the simulation.

#### Experimental evaluation of the simulator

After tuning the simulation, as explained in “Parameter tuning” section, we asked the same two neurosurgeons who already participated in the force/torque quantification experiments (see “Force and torque quantification” section) to palpate the virtual pedicle screw tract with our simulator (see Fig. 4). Both surgeons performed the palpation four times. We additionally tried two alternative parameter sets, the results of which are provided in Online Resource 1. The position and orientation of the haptic device end-effector, the rendered forces and torques, and the position of the virtual pedicle probe tip were recorded at the update rate of the simulation (roughly 1–1.8 kHz) for all palpations.

### Results

#### Simulation of recorded trajectories

In an offline simulation of the recorded motion of the force/torque sensor during palpation, our simulator computed the forces and torques shown in Fig. 2.

#### Experimental evaluation of simulator

**Fig. 5:**
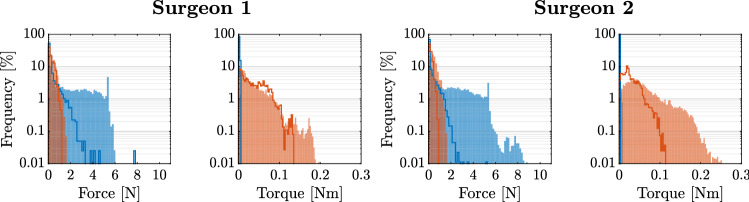
Distribution of the forces and torques sent to the haptic device during the simulated palpation task using the tuned parameters. The blue histograms visualize the computed forces along the longitudinal axis of the pedicle probe handle and the twisting torques. The orange histograms visualize the computed forces in a plane perpendicular to the longitudinal axis of the pedicle probe handle and the bending torques. The histograms for each surgeon comprise the data of 4 trials. For comparison, the measured force and torque distributions during the palpations of the boar vertebra with the instrumented pedicle probe are shown with solid lines

The distribution of the computed forces and torques sent to the haptic device for rendering, while the surgeons palpated the virtual pedicle screw tract are displayed in Fig. 5.

A plot of the motion of the virtual probe and the corresponding forces and torques computed by the simulation are provided in Online Resource 1 for an exemplary trial. The mean, standard deviation, and maximum of all computed forces and torques above 0.01 N and 0.001 Nm, respectively, are shown in Table 2.

**Table 2:** Mean, standard deviation, and maximum of the computed forces and torques during palpation of the virtual vertebra screw tract using the tuned parameters

	$F_{xy}$ (N)			$F_{z}$ (N)			$T_{xy}$ (Nm)			$T_{z}$ (Nm)		
	$\mu$	$\sigma$	max	$\mu$	$\sigma$	max	$\mu$	$\sigma$	max	$\mu$	$\sigma$	max
Surgeon 1	0.39	0.28	1.54	1.91	1.79	9.40	0.044	0.033	0.216	< 0.001	< 0.001	< 0.001
Surgeon 2	0.49	0.28	1.63	1.88	1.76	10.60	0.064	0.043	0.286	< 0.001	< 0.001	< 0.001

Forces below 0.01 N and torques below 0.001 Nm have not been considered

## Discussion

With our first experiment we were able to quantify the forces and torques prevalent while palpating a real pedicle screw tract and observe how the forces and torques change depending on which part of the screw tract was examined. In the first part of the trial shown in Fig. 2, a sweeping motion along the longitudinal (*z*) axis of the probe and pronounced changes in the angle between the probe and the screw tract can be recognized. This indicates that the surgeon was focusing on palpating the sides of the screw tract, which mainly resulted in forces perpendicular to the probe ($F_{xy}$) and bending torques ($T_{xy}$). Subsequently, the surgeon was focusing on verifying the integrity of the anterior wall of the screw tract by tapping against it several times, discernible by the short motions in longitudinal direction near the end of the screw tract. This technique resulted in multiple distinct peaks of the longitudinal forces $F_{z}$ in quick succession, and low forces and torques otherwise.

Using the recorded motion data from an exemplary palpation as an input for our simulator resulted in simulated forces and torques that were similar to the recorded ones with respect to their temporal progression and magnitude (see Fig. 2).

The discrepancies that were observed between the measured and computed forces and torques could be due to small differences in the mechanical properties and bending behavior of the simulated and the real probe or to inaccuracies in the measurement of the pedicle probe position relative to the vertebra position. The high-frequency components in the computed forces and torques could be caused by the fact that during the offline simulation the virtual probe always followed the recorded path of the real probe, irrespective of the resistive forces computed along this path. Also, considering the voxel spacing of $69.5\,\upmu \hbox {m}$, small inaccuracies in the motion capture data can result in an offset of the probe tip by several voxels. This in turn could cause the tip of the virtual probe to be slightly pushed into the bony tissue, while the real probe was just touching the surface. When using the haptic device instead of a simple offline simulation this is less of a problem because the device handle and the user’s hand would be pushed back by the generated forces. Moreover, during a simulation with the haptic device, the forces computed by our algorithm would be additionally dampened by the rate limit of the device motors and the inertia of the device mechanics. The several positive peaks in $F_{z}$ following the examination of the anterior wall (around the 7th second in Fig. 2) were most likely caused by the tip of the virtual probe penetrating the bony tissue and temporarily getting stuck in an area of the CT scan corresponding to porous structures of the bone.

When the surgeons tested our simulator, the mean forces perpendicular to the probe and the mean bending torques computed by the simulator were about 2–3 times higher than the ones measured with the real probe (see Tables 1 and 2). The mean forces along the longitudinal axis of the probe were about 6–8 times higher than the ones measured with the real probe. We assume that this was partly due to the tip of the virtual probe penetrating the surface of the virtual screw tract due to inaccuracies in the haptic simulation and therefore getting stuck in the porous structure of the simulated bone. This, in turn, led to occasional oscillations of the tip between two opposite bone structures. The higher mean forces and torques could also have originated from a different palpation behavior of the surgeons using the simulator compared to a real pedicle probe. Such a behavioral change could partly be due to the surgeons not being accustomed to interacting with a haptic device. Additionally, the imperfect, albeit high device transparency could have caused the surgeons to interact with the haptic device handle more heavy-handedly than with the real probe. This could entail that the interaction forces and torques may have to be tuned to the perception of expert surgeons rather than to the measured forces and torques with the real probe.

The purpose of this work was to show the technical feasibility of our direct volume rendering approach for the simulation of pedicle screw tract palpation. Thus, for an initial evaluation of our approach, we implemented the normative case, i.e., a correctly drilled screw tract. We could show that the determined requirements can be fulfilled by our approach and that direct volume rendering can be an alternative to mesh- or surface-based simulators. However, in a clinical setting, pedicle screw tracts with anterior, medial, lateral, superior, or inferior breaches need to be differentiated from intact screw tracts. Our approach theoretically allows simulating such a setting as well as the drilling process itself by removing voxels along the drilling path and periodically recomputing the force field around the modified area of the voxel data. However, the implementation of these features and a targeted training study are required to evaluate the effectiveness of our approach in training surgeons to correctly drill screw tracts and detect clinically relevant pedicle breaches.

The number of haptic interaction points may also have to be re-evaluated for data sets with pedicle breaches. Using more interaction points can improve the achieved haptic detail at the cost of more expensive computation. We believe that the chosen number of three interaction points could provide acceptable results for breach detection, but a more realistic experience could possibly be achieved with additional interaction points around the probe tip to better represent its spherical shape.

For the work presented here, we used a young boar vertebra. Pig bone was found to have a high structural and compositional similarity to human bone [18]. Wild boar bone shows certain geometric and structural differences compared to domestic pigs [15], but it most likely does not come with the disadvantages of commercial pig breeds such as large growth rates and excessive body weight [18]. Given that patients undergoing pedicle screw placement surgeries range from young children to elderly [11, 20], the age of the boar vertebrae is most likely representing at least part of the potential patient population. In conclusion, we expect that similar results would be obtained with human vertebrae for the quantification of the prevalent forces and torques during pedicle screw tract palpation.

## Conclusion

We demonstrated the feasibility of direct visual and haptic volume rendering to simulate the palpation of pedicle screw tracts. Our approach of fine-tuning the simulation by measuring the forces and torques that are predominant while palpating a real vertebra produced promising results. The potential benefit of using our simulator in training surgeons to correctly identify pedicle breaches in a clinical setting has to be investigated in a future study. However, our approach proved to be suitable for surgical simulation and opens the door for patient-specific training of planned interventions.

## Electronic supplementary material

Below is the link to the electronic supplementary material.

Supplementary material 1 (pdf 1222 KB)

Supplementary material 2 (mp4 56010 KB)

## References

[1] Aoude AA, Fortin M, Figueiredo R, Jarzem P, Ouellet J, Weber MH (2015). Methods to determine pedicle screw placement accuracy in spine surgery: a systematic review. Eur Spine J.

[2] Avila RS, Sobierajski LM (1996) A haptic interaction method for volume visualization. In: Proceedings of seventh annual IEEE visualization’96. IEEE, pp 197–204

[3] Breese R, Piazza M, Quinsey C, Blatt J (2020). Tactile skill-based neurosurgical simulators are effective and inexpensive. World Neurosurg.

[4] Castro WH, Halm H, Jerosch J, Malms J, Steinbeck J, Blasius S (1996). Accuracy of pedicle screw placement in lumbar vertebrae. Spine.

[5] Donohue ML, Moquin RR, Singla A, Calancie B (2014). Is in vivo manual palpation for thoracic pedicle screw instrumentation reliable?. J Neurosurg Spine.

[6] Faludi B, Zoller EI, Gerig N, Zam A, Rauter G, Cattin PC (2019) Direct visual and haptic volume rendering of medical data sets for an immersive exploration in virtual reality. In: International conference on medical image computing and computer-assisted intervention. Springer, pp 29–37

[7] Gang C, Haibo L, Fancai L, Weishan C, Qixin C (2012). Learning curve of thoracic pedicle screw placement using the free-hand technique in scoliosis: how many screws needed for an apprentice?. Eur Spine J.

[8] Gautschi OP, Schatlo B, Schaller K, Tessitore E (2011). Clinically relevant complications related to pedicle screw placement in thoracolumbar surgery and their management: a literature review of 35,630 pedicle screws. Neurosurg Focus.

[9] Gelalis ID, Paschos NK, Pakos EE, Politis AN, Arnaoutoglou CM, Karageorgos AC, Ploumis A, Xenakis TA (2012). Accuracy of pedicle screw placement: a systematic review of prospective in vivo studies comparing free hand, fluoroscopy guidance and navigation techniques. Eur Spine J.

[10] Gonzalvo A, Fitt G, Liew S, de la Harpe D, Turner P, Ton L, Rogers MA, Wilde PH (2009). The learning curve of pedicle screw placement: how many screws are enough?. Spine.

[11] Kim YJ, Lenke LG, Bridwell KH, Cho YS, Riew KD (2004). Free hand pedicle screw placement in the thoracic spine: is it safe?. Spine.

[12] Kosmopoulos V, Schizas C (2007). Pedicle screw placement accuracy: a meta-analysis. Spine.

[13] Laycock SD, Day A (2007) A survey of haptic rendering techniques. In: Computer graphics forum, vol 26. Wiley Online Library, pp 50–65

[14] Lehman RA, Potter BK, Kuklo TR, Chang AS, Polly DW, Shawen SB, Orchowski JR (2004). Probing for thoracic pedicle screw tract violation (s): is it valid?. Clin Spine Surg.

[15] Mainland I, Schutkowski H, Thomson AF (2007). Macro-and micromorphological features of lifestyle differences in pigs and wild boar. Anthropozoologica.

[16] Malone HR, Syed ON, Downes MS, D’Ambrosio AL, Quest DO, Kaiser MG (2010). Simulation in neurosurgery: a review of computer-based simulation environments and their surgical applications. Neurosurgery.

[17] Mason A, Paulsen R, Babuska JM, Rajpal S, Burneikiene S, Nelson EL, Villavicencio AT (2014). The accuracy of pedicle screw placement using intraoperative image guidance systems: a systematic review. J Neurosurg Spine.

[18] Pearce AI, Richards RG, Milz S, Schneider E, Pearce SG (2007). Animal models for implant biomaterial research in bone: a review. Eur Cell Mater.

[19] Proietti T, Crocher V, Roby-Brami A, Jarrasse N (2016). Upper-limb robotic exoskeletons for neurorehabilitation: a review on control strategies. IEEE Rev Biomed Eng.

[20] Ranade A, Samdani AF, Williams R, Barne K, McGirt MJ, Ramos G, Betz RR (2009). Feasibility and accuracy of pedicle screws in children younger than eight years of age. Spine.

[21] Ren J, Patel RV, McIsaac KA, Guiraudon G, Peters TM (2008). Dynamic 3-D virtual fixtures for minimally invasive beating heart procedures. IEEE Trans Med Imaging.

[22] Ruikar DD, Hegadi RS, Santosh K (2018). A systematic review on orthopedic simulators for psycho-motor skill and surgical procedure training. J Med Syst.

[23] Sedory DM, Crawford JJ, Topp RF (2011). The reliability of the ball-tipped probe for detecting pedicle screw tract violations prior to instrumenting the thoracic and lumbar spine. Spine.

[24] Tian NF, Huang QS, Zhou P, Zhou Y, Wu RK, Lou Y, Xu HZ (2011). Pedicle screw insertion accuracy with different assisted methods: a systematic review and meta-analysis of comparative studies. Eur Spine J.

[25] Verma R, Krishan S, Haendlmayer K, Mohsen A (2010). Functional outcome of computer-assisted spinal pedicle screw placement: a systematic review and meta-analysis of 23 studies including 5,992 pedicle screws. Eur Spine J.

[26] Vieweg U, Grochulla F (2012). Manual of spine surgery.

