# Supplementary material for: Force quantification and simulation of pedicle screw tract palpation using direct visuo-haptic volume rendering
Source: Int J Comput Assist Radiol Surg. 2020 Sep 21;15(11):1797–805. doi: 10.1007/s11548-020-02258-0 (PMC7603448; doi:10.1007/s11548-020-02258-0)
Supplement: Supplementary file 1 — Supplementary material 1 (pdf 1222 KB) [file 11548_2020_2258_MOESM1_ESM.pdf]

Force quantification and simulation of pedicle screw tract palpation using direct visuo-haptic volume rendering

Supplementary Material

Esther I. Zoller[1] · Balázs Faludi[2] · Nicolas Gerig[1] · Gregory F. Jost[3] · Philippe C. Cattin[2] · Georg Rauter[1]

CT data processing

Reconstructing the force sensor pose

In the vertebra CT, we determined the center of all four motion capture markers by fitting a sphere to 12 points selected on the surface of the marker sphere. This allowed us to determine the coordinate system of the marker cluster and the transformation between the CT coordinate system and the marker cluster coordinate system. Additionally, we determined the positions of the screw tract entrance and end in the vertebra CT. From this, we computed the axis of the screw tract and arbitrarily defined the remaining two axes as the screw tract is rotationally symmetric. Similarly, in the probe CT, we determined the coordinate system of the marker cluster and the pose of the force/torque sensor. Given the motion tracking data and the above-mentioned measurements, we could then determine the pose of the force/torque sensor relative to the screw tract.

Haptic rendering

As explained in the "Haptic rendering" section in the paper, we do not use an explicit definition of collision surfaces to compute the haptic force feedback. Instead, our method starts rendering a repulsive force at a short distance to visible voxels and smoothly increases the magnitude of the rendered force as the haptic interaction point gets closer. To achieve this, we applied a Gaussian blur to the voxel opacity values. This reduces the slope of the voxel opacity gradient at tissue borders and results in a smoother increase of forces. We empirically found a blur kernel with a size of 15 voxels and $\sigma = 1$ voxel in all three dimensions to provide an acceptable compromise between keeping the bone tissue stiffness within the capabilities of our haptic device and losing too much haptic detail. The haptic forces correspond to the negative gradient of the blurred voxel opacity values. Figure 1 shows a slice of the raw CT data, the same slice with the transfer function applied and the result after applying the blur kernel.

Simulation of pedicle screw tract palpation

Exemplary sample of the computed forces/torques during simulation

The motion of the pedicle probe handle relative to the pedicle screw tract in the vertebra and the corresponding computed forces/torques are shown in Fig. 2 for an excerpt of an exemplary trial. Oscillations can be observed at around $t = 3s$ in Fig. 2. As described in the "Discussion" section of the paper, we assume that these oscillations were due to the virtual probe getting stuck in the porous structure of the bone.

Alternative simulation parameter sets

As the first surgeon reported that the rendered forces and torques felt rather low with the simulation parameters resulting from the tuning (P1: $s_f = 0.3,\ s_t = 0.3$), we additionally tried changing the force and torque scaling factors to P2: $s_f = 0.3,\ s_t = 1.0$ and P3: $s_f = 1.0,\ s_t = 1.0$ for one palpation each.

Esther I. Zoller and Balázs Faludi have contributed equally to this work.

Esther I. Zoller
esther.zoller@unibas.ch

Balázs Faludi
balazs.faludi@unibas.ch

[1] BIROMED-Lab, Department of Biomedical Engineering, University of Basel, Basel, Switzerland
[2] CIAN, Department of Biomedical Engineering, University of Basel, Basel, Switzerland
[3] Spinale Chirurgie, Spitalzentrum Biel, Biel, Switzerland

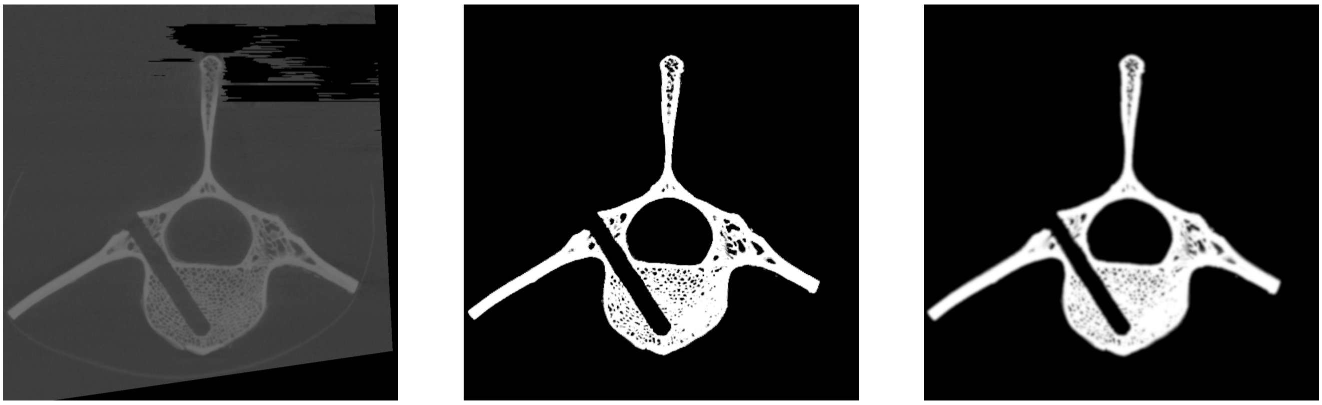

(a) (b) (c)

Fig. 1: a) A slice from the nanotom CT of the boar vertebra that was used for the force/torque quantification measurements as well as for the simulation. The black area at the top right is where the motion capture marker cluster was masked from the data set. b) The voxel opacities of the same slice after applying the transfer function. c) The result after applying the Gaussian blur kernel with a size of 15 voxels and $\sigma = 1$ voxel.

The distribution of the computed forces and torques sent to the haptic device for rendering while the surgeons tested the simulation with all three parameter sets are displayed in Figure 3. The mean, standard deviation, and maximum of all measured and computed forces and torques above 0.01 N and 0.001 Nm, respectively, are shown in Table 1, separated by surgeon and condition.

Nano17 vs. Mini45 force sensor

For the measurements reported in the paper, we used a 6-axis Nano17 force/torque sensor (ATI Industrial Automation, Apex, NC, USA) with SI-12-0.12 calibration. However, since we were unsure about the maximum torques to be expected during pedicle probing, we also conducted the measurements with a 6-axis Mini45 force/torque sensor (ATI Industrial Automation, Apex, NC, USA) with SI-580-20 calibration for the first surgeon. The Mini45 has a much higher sensing range compared to the Nano17, but also a lower resolution and a higher mass (91.7 g compared to 9.07 g) that might distort the measurements. Thus, after completing the reported measurements with the Nano17 force/torque sensor, we exchanged the sensor and repeated the measurements with the Mini45 force/torque sensor. Initial inspection of the recorded data showed a very small signal to noise ratio for the Mini45. Additionally, the surgeon mentioned that the probe with the Mini45 felt unnaturally heavy. Our doubts regarding the sensing ranges of the Nano17 proved to be unfounded, as the surgeons applied lower forces and torques than we expected. Therefore, we decided to disregard the data collected with the Mini45 and used only the Nano17 for the measurements with the second surgeon.

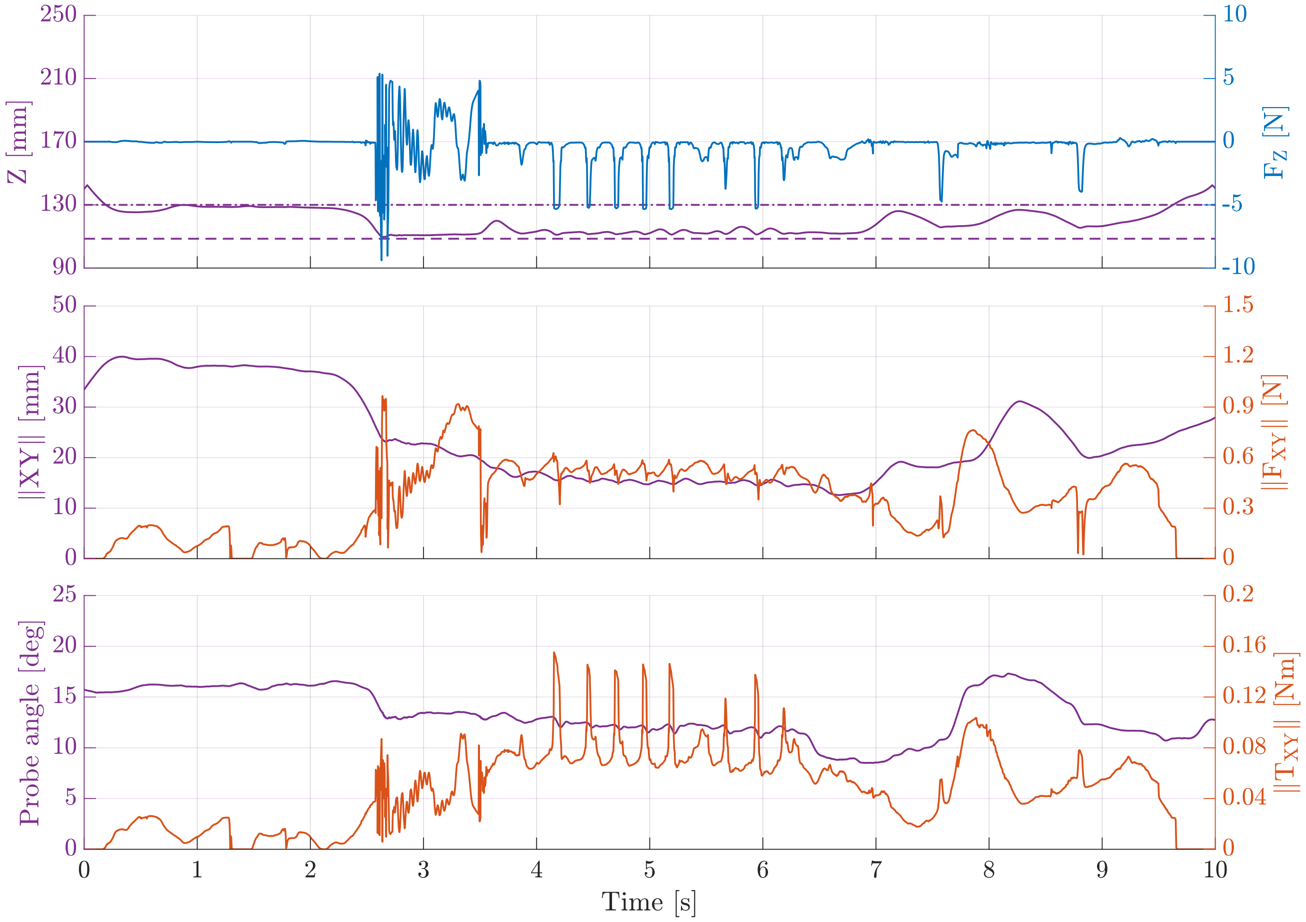

Fig. 2: Computed force/torque data from an exemplary simulated palpation trial of the more experienced surgeon (surgeon 1). For better visualization, only an excerpt of the trial is shown. *Top:* Distance between the probe shank-handle interface (where the force/torque sensor was mounted in the real probe) and the screw tract entrance along the screw tract axis (purple) and the computed longitudinal forces (blue). The horizontal lines indicate where the pedicle probe tip enters (dash-dot) and reaches the anteriormost end (dashed) of the screw tract. *Middle:* Distance from the probe shank-handle interface to the screw tract axis (purple) and the computed transverse forces (orange). *Bottom:* Angle between screw tract and probe handle (purple) and the computed bending torques (orange).

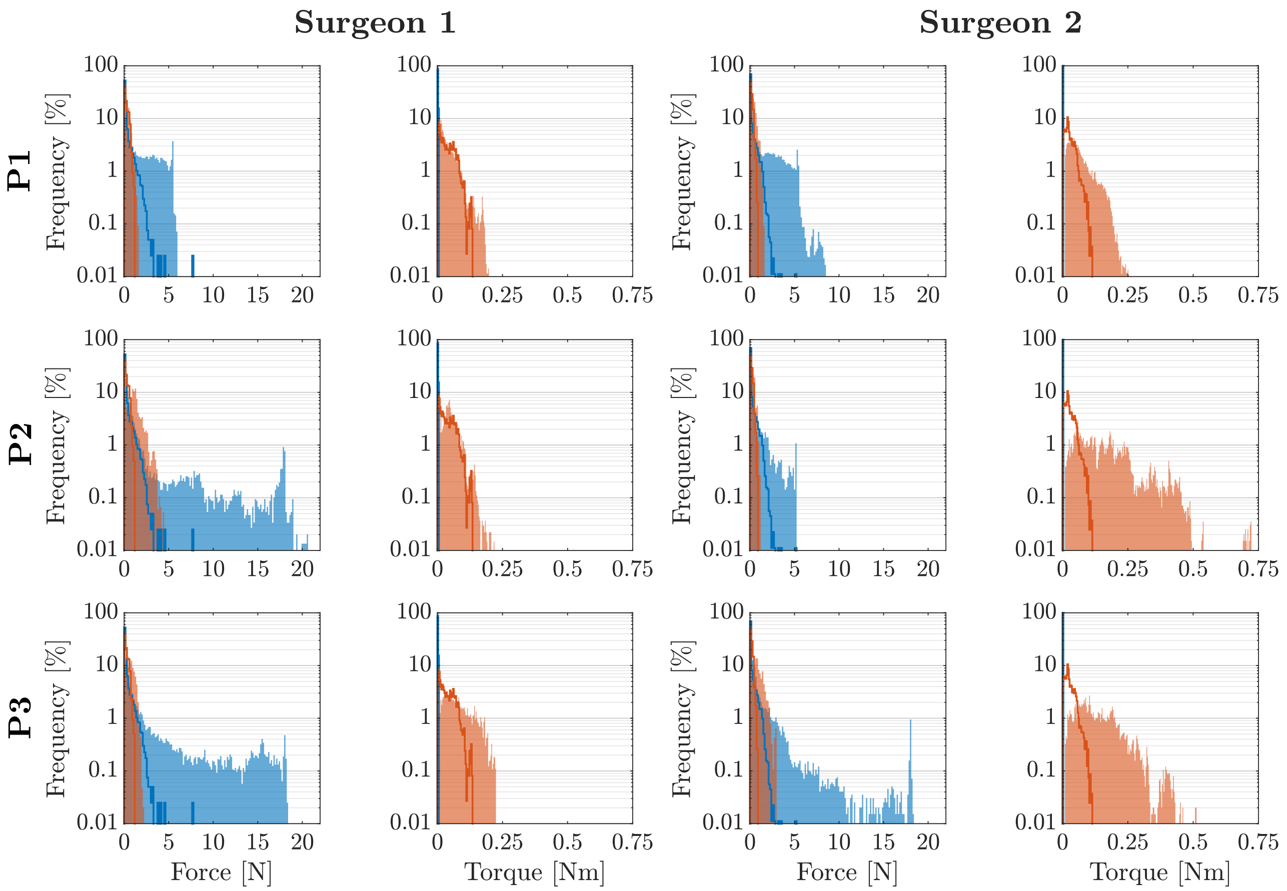

Fig. 3: Distribution of the forces and torques sent to the haptic device for rendering during the simulated palpation task. The blue histograms visualize the computed forces along the longitudinal axis of the pedicle probe handle and the twisting torques. The orange histograms visualize the computed forces in a plane perpendicular to the longitudinal axis of the pedicle probe handle and the bending torques. For comparison, the measured force and torque distributions during the palpations of the boar vertebra with the instrumented pedicle probe are shown with solid lines. The first row displays the calculated forces and torques with the parameters P1: $s_f = s_t = 0.3$, as determined during the parameter tuning. The data visualized in this row are based on 4 measurements per surgeon. The second row displays the forces and torques obtained with P2: $s_f = 0.3$ and $s_t = 1.0$. The data visualized in this row are based on 1 measurement per surgeon. The forces and torques obtained for P3: $s_f = s_t = 1.0$ are shown in the last row. The data visualized in this row are based on 1 measurement per surgeon.

Table 1: Mean, standard deviation, and maximum of the measured and computed forces and torques during palpation of the vertebra screw tract, separated by surgeon and condition. Forces below 0.01 N and torques below 0.001 Nm have not been considered. M stands for the data measured with the real pedicle probe. P1, P2, P3 stand for the simulation parameters $s_f = s_t = 0.3$, $s_f = 0.3$ and $s_t = 1.0$, and $s_f = s_t = 1.0$, respectively.

		F_{xy} [N]			F_z [N]			T_{xy} [Nm]			T_z [Nm]		
		μ	σ	max	μ	σ	max	μ	σ	max	μ	σ	max
M	Surgeon 1	0.28	0.24	1.12	0.31	0.47	7.78	0.037	0.029	0.134	0.002	< 0.001	0.005
	Surgeon 2	0.19	0.15	0.89	0.23	0.36	5.14	0.028	0.020	0.113	0.001	< 0.001	0.002
P1	Surgeon 1	0.39	0.28	1.54	1.91	1.79	9.40	0.044	0.033	0.216	< 0.001	< 0.001	< 0.001
	Surgeon 2	0.49	0.28	1.63	1.88	1.76	10.60	0.064	0.043	0.286	< 0.001	< 0.001	< 0.001
P2	Surgeon 1	1.21	0.75	4.42	2.16	4.21	22.01	0.053	0.029	0.253	< 0.001	< 0.001	< 0.001
	Surgeon 2	0.35	0.22	1.16	0.66	1.05	5.24	0.160	0.111	0.722	< 0.001	< 0.001	< 0.001
P3	Surgeon 1	0.68	0.42	2.25	2.50	4.36	20.43	0.082	0.050	0.223	< 0.001	< 0.001	< 0.001
	Surgeon 2	0.95	0.56	2.98	1.38	2.74	18.36	0.132	0.080	0.512	< 0.001	< 0.001	< 0.001
